# Recruitment and Results Reporting of COVID-19 Randomized Clinical Trials Registered in the First 100 Days of the Pandemic

**DOI:** 10.1001/jamanetworkopen.2021.0330

**Published:** 2021-03-01

**Authors:** Perrine Janiaud, Cathrine Axfors, John P. A. Ioannidis, Lars G. Hemkens

**Affiliations:** 1 Department of Clinical Research, University of Basel, Basel, Switzerland; 2 Meta-Research Innovation Center at Stanford, Stanford University, Stanford, California

## Abstract

This survey analysis of randomized clinical trials registered within 100 days of the first reported case of coronavirus disease 2019 assessed recruitment and results reporting.

## Introduction

The coronavirus disease 2019 (COVID-19) pandemic caused an unprecedented drive to launch clinical trials.^1^ We assessed the recruitment and results reporting of randomized clinical trials (RCTs) to treat or prevent COVID-19 registered within 100 days of the first case reported to the World Health Organization.^1^

## Methods

All RCTs registered in ClinicalTrials.gov and the World Health Organization International Clinical Trials Registry Platform between January 1 and April 9, 2020, were included. These trials had been previously identified in a systematic assessment.^1^ Between October 5 and 15, 2020, using contact information provided in the registries, we sent emails to all trial groups, requesting information on recruitment and enrollment status, reasons for trial termination or discontinuation, and any results reporting. We assessed whether trials recruited 75% or more of their target sample size, stopped before 75% recruitment, or were continuing to recruit (on schedule or delayed) but not yet 75% recruited. On October 19, we searched the Living Overview of Evidence platform^2^ for results posted as preprints, published in peer-reviewed journals, or reported in trial registries. The survey results were used to complement the information from our search. Associations of trial characteristics—location (ie, China vs elsewhere), planned sample size greater or less than the median (114 participants [interquartile range, 70-334 participants]), registered on or after the median registration date (March 15), and type of control group—with recruitment and results reporting were explored using logistic regressions. We used R version 3.6.2 (R Project for Statistical Computing). The *P* value threshold was <.05 in 2-tailed tests. Institutional review board approval and an informed consent waiver were not required for this study because the aggregated trial data collected are not subject to the health regulations of Switzerland.

## Results

There were 516 trials identified. Seventy-six trial teams (14.7%) responded to the survey, and we found results reported for 53 trials (10.3%). Overall, 56 trials (10.8%) were completed, 24 (4.6%) terminated early, 111 (21.5%) were ongoing on schedule, 126 (24.4%) were ongoing but delayed, 39 (7.6%) were ongoing without reporting an expected end date, 155 (30%) had not started or were discontinued, and 5 (1.0%) had unknown status.

Recruitment had started in 361 trials (70.0%). Of 108 started trials with detailed recruitment information obtained from investigators and/or reported results, 38 (35.1%) reached at least 75% of their target sample size, 34 (31.5%) stopped before 75%, and 36 (33.3%) had not yet recruited 75% of their target. Median (interquartile range [IQR]) recruitment was 45.2% (19.6%-100%). Trials were less likely to begin recruitment when they were less than vs greater than median sample size (152/250 [60.8%] vs 203/256 [79.3%]; adjusted odds ratio [OR], 0.60; 95% CI, 0.39-0.92; *P* = .02) or when they were registered in China vs elsewhere (152/267 [56.2%] vs 204/242 [84.3%]; adjusted OR, 0.32; 95% CI, 0.17-0.61; *P* < .001) (Table).

**Table.** Characteristics of Randomized Clinical Trials in the First 100 Days of the Coronavirus Disease 2019 Pandemic Starting Recruitment and Reporting Results

Characteristics	RCTs that started recruitment^a^					RCTs reporting results				
	No./total No. (%)^a^	Bivariable OR (95% CI)	*P* value	Multivariable adjusted OR (95% CI)	*P* value	No./total No. (%)	Bivariable OR (95% CI)	*P* value	Multivariable adjusted OR (95% CI)	*P* value
Country^b^										
Elsewhere	204/242 (84.3)	1 [Reference]	NA	1 [Reference]	NA	28/244 (11.5)	1 [Reference]	NA	1 [Reference]	NA
China	152/267 (56.2)	0.24 (0.16-0.37)	<.001	0.32 (0.17-0.61)	<.001	25/270 (9.3)	0.78 (0.44-1.39)	.41	0.24 (0.10-0.62)	.003
Sample size target^c^										
>144 participants	203/256 (79.3)	1 [Reference]	NA	1 [Reference]	NA	33/256 (12.9)	1 [Reference]	NA	1 [Reference]	NA
<144 participants	152/250 (60.8)	0.40 (0.27-0.60)	<.001	0.60 (0.39-0.92)	.02	20/253 (7.9)	0.58 (0.32-1.03)	.07	0.63 (0.33-1.21)	.17
Registration^d^										
On or after March 15, 2020	208/258 (80.6)	1 [Reference]	NA	1 [Reference]	NA	20/259 (7.7)	1 [Reference]	NA	1 [Reference]	NA
Before March 15, 2020	146/250 (58.4)	0.34 (0.22-0.5)	<.001	0.86 (0.47-1.59)	.64	33/252 (13.1)	1.8 (1.01-3.28)	.05	6.08 (2.50-14.99)	<.001
Control type										
Standard of care	187/277 (67.5)	1 [Reference]	NA	1 [Reference]	NA	28/281 (10.0)	1 [Reference]	NA	1 [Reference]	NA
Placebo	92/128 (71.9)	1.23 (0.78-1.96)	.38	0.88 (0.53-1.48)	.64	13/129 (10.1)	1.01 (0.49-1.99)	.97	1.02 (0.47-2.09)	.97
Active	77/106 (72.6)	1.28 (0.78-2.12)	.33	1.05 (0.62-1.8)	.85	12/106 (11.3)	1.15 (0.54-2.31)	.70	1.17 (0.54-2.41)	.67

Abbrevations: NA, not applicable; OR, odds ratio; RCT, randomized clinical trial.

^a^ Five RCTs had an unknown recruitment status, leaving a sample size of 511.

^b^ Argentina (1 trial); Australia (4); Austria (1); Belgium (6); Brazil (7); Canada (11); China (267); Colombia (1); Czech Republic (1); Denmark (6); Egypt (1); France (30); Germany (7); Greece (2); Hong Kong (2); India (1); multinational (22); Iran (8); Iraq (1); Ireland (2); Israel (3); Italy (5); Japan (1); Republic of Korea (3); Malaysia (1); Mexico (5); Netherlands (9); New Zealand (1); Norway (4); Pakistan (3); Poland (1); Russian Federation (2); Saudi Arabia (1); Singapore (1); Spain (21); Switzerland (4); Thailand (1); United Kingdom (7); United States (56); and Vietnam (1). Two RCTs had missing values for country.

^c^ Seven RCTs had missing values for sample size target.

^d^ Median (interquartile range) registration date was March 15, 2020 (February 18 to March 31, 2020), and 5 RCTs had missing values for registration dates.

Of the 53 trials with reported results, 13 (24.5%) were posted as preprints, 39 (73.6%) were published in peer-reviewed journals, and 1 (1.9%) was posted on a registry. Results reporting was less likely for trials from China vs elsewhere (25/270 [9.3%] vs 28/244 [11.5%]; adjusted OR, 0.24; 95% CI, 0.10-0.62; *P* = .003) but more likely for trials registered before the median registration date (March 15, 2020) vs later (33/252 [13.1%] vs 20/259 [7.7%]; adjusted OR, 6.08; 95% CI, 2.5-14.99; *P* < .001). Type of control group was not associated with start of recruitment or results reporting.

Of the 24 terminated and 46 discontinued trials, investigators of 14 RCTs communicated the reasons for stopping. Most frequently, trials were stopped because of decreasing COVID-19 cases (8 trials [57.1%]) and emerging data raising concerns of safety (6 trials [42.9%]) or futility (2 trials [14.3%]).

## Discussion

Overall, 30% of COVID-19 RCTs initiated in the first 100 days of the pandemic did not begin recruitment, and only 10% had results reported by mid-October, suggesting the possibility of substantial research waste.^3^ Delayed completion may be explained for some countries, such as China, by slow or halted recruitment due to decreasing COVID-19 cases after the first wave.

This study is limited by a low response rate to the survey and by unknown results because of inconsistent updates of trial registries and delayed publications. Successfully conducting a trial during a pandemic is challenging. Nevertheless, collaborative efforts such as consortiums of trials prospectively planning to pool their results^4^ and adaptive platform trials such as the RECOVERY trial^5^ are promising approaches to provide reliable and timely evidence.
